# Choriocarcinoma Presenting as a Pleural Effusion

**DOI:** 10.7759/cureus.9667

**Published:** 2020-08-11

**Authors:** Leonard Hamera, Marie-Louise Posch, Sunoj Abraham, Jeffrey Jordan

**Affiliations:** 1 Internal Medicine, Citrus Memorial Hospital, Inverness, USA; 2 Pulmonology/Critical Care, Citrus Memorial Hospital, Inverness, USA; 3 Internal Medicine, HCA-USF Consortium, Citrus Memorial Hospital, Inverness, USA

**Keywords:** lung mass, choriocarcinoma, germ cell tumor, mediastinal tumor, pleural effusion

## Abstract

Choriocarcinoma is a germ cell tumor characterized by widespread metastases and poorly differentiated cells. Non-gestational choriocarcinoma, or primary choriocarcinoma is a trophoblastic disease which is associated with a poor patient prognosis and is markedly angioinvasive. Primary non-gestational mediastinal choriocarcinoma is a very rare disease and represents an aggressive malignancy, primarily seen in young males. Those with primary mediastinal choriocarcinoma have symptoms that are non-specific such as cough, dyspnea, hemoptysis, and chest pain.

Here we present the case of a 47-year-old Caucasian female who presented with worsening dyspnea and cough. Laboratory testing revealed elevated alkaline phosphatase, human chorionic gonadotropin, and cancer antigen 125. Chest X-ray was significant for a large right pleural effusion and a computed tomography angiogram of the chest showed a soft tissue mass in the anterior medial right lung base/right middle lobe. Thoracentesis yielded results consistent with malignant cells favoring a germ cell tumor. Biopsy of the mediastinal mass revealed positivity for inhibin and both human chorionic gonadotropin and CD-10 which led to the diagnosis of primary choriocarcinoma.

Primary mediastinal choriocarcinoma is uncommon and often has a non-specific clinical presentation. A high degree of suspicion is needed as this malignancy can be aggressive, necessitating urgent definitive tissue biopsy diagnosis to guide appropriate therapy.

## Introduction

Choriocarcinoma is a nonseminomatous germ cell tumor with poorly differentiated cells. It is divided into gestational and non-gestational subtypes. In gestational trophoblastic neoplasia (GTN), choriocarcinoma is the most aggressive histologic type and frequently metastasizes to the lungs [1]. Non-gestational choriocarcinoma, or primary choriocarcinoma (PCC), however, is a considerably uncommon trophoblastic disease which is associated with a poor patient prognosis and is markedly angioinvasive. It is by definition without a primary tumor in the ovaries or testes and does not include metastatic disease to the retroperitoneal lymph nodes.

Primary nonseminomatous mediastinal choriocarcinoma (PNMC) is a distinctly rare disease that represents an aggressive malignancy. While exact numbers are unknown, some have postulated that the incidence of extragonadal germ cell tumors is between 1.8 to 3.4/1 million with germ cell tumors in general accounting for up to 16% of mediastinal tumors [2, 3]. PCC has been reported in the central nervous system, pineal body and suprasellar regions, mediastinum, retroperitoneum, lung and liver. Additionally, although PCC is very rare in males, the primary mediastinal choriocarcinoma variant is almost exclusively seen in young males [4, 5]. Those with primary mediastinal choriocarcinoma have symptoms that are non-specific and share features seen in metastatic disease which makes the diagnosis difficult. Most commonly, symptoms reported include cough, dyspnea, hemoptysis, and chest pain [4, 6]. Like other choriocarcinoma subtypes, elevated serum beta-human chorionic gonadotropin (hCG) levels as well as trophoblastic cells on histologic examination support the diagnosis. Invasive moles and choriocarcinomas can produce copious amounts of hCG, ranging from 100 to greater than 100,000 MIU/mL [7]. Placental site trophoblastic tumors and epithelioid trophoblastic tumors on the other hand produce low levels of hCG, less than 1000 MIU/mL [8]. Of the women affected, presenting problems related to hormonal irregularities such as change in menstrual pattern or amenorrhea were reported. Gynecomastia and other signs of feminization in men have also been documented, but these are atypical [9].

It is still unclear as to how PCC develops, but it has been postulated that it may be the result of a metastasis from a primary gonadal choriocarcinoma that spontaneously regressed. It may also take its origin from retained primordial germ cells that, during embryogenesis, abnormally migrate [9].

## Case presentation

A 47-year-old Caucasian female presented to the emergency department for worsening shortness of breath. She described a three-month history of dyspnea on exertion, difficulty with deep breathing, dry cough, and right-sided pleuritic chest pain. Symptoms gradually worsened until three days prior to presentation when she started having symptoms at rest. Pertinent negative findings included no fevers, chills, upper respiratory symptoms, hemoptysis, orthopnea, swelling, additional chest pain, or weight changes. Her past medical history was significant for menorrhagia and uterine ablation 15 years ago. Family history was notable for a brother and mother with multiple sclerosis and a father who passed away from gastric cancer. She denied smoking, alcohol use, recreational drug use, sick contacts, or recent travel. Vital signs were remarkable for tachycardia with a rate of 114 beats per minute, tachypnea of 22 breaths per minute and hypoxia of 87% oxygen saturation on room air that improved to 92% on 2 liters of supplemental oxygen. Pertinent physical exam findings included decreased breath sounds on the right lower lung fields and dullness to percussion over the same area.

**Figure 1:**
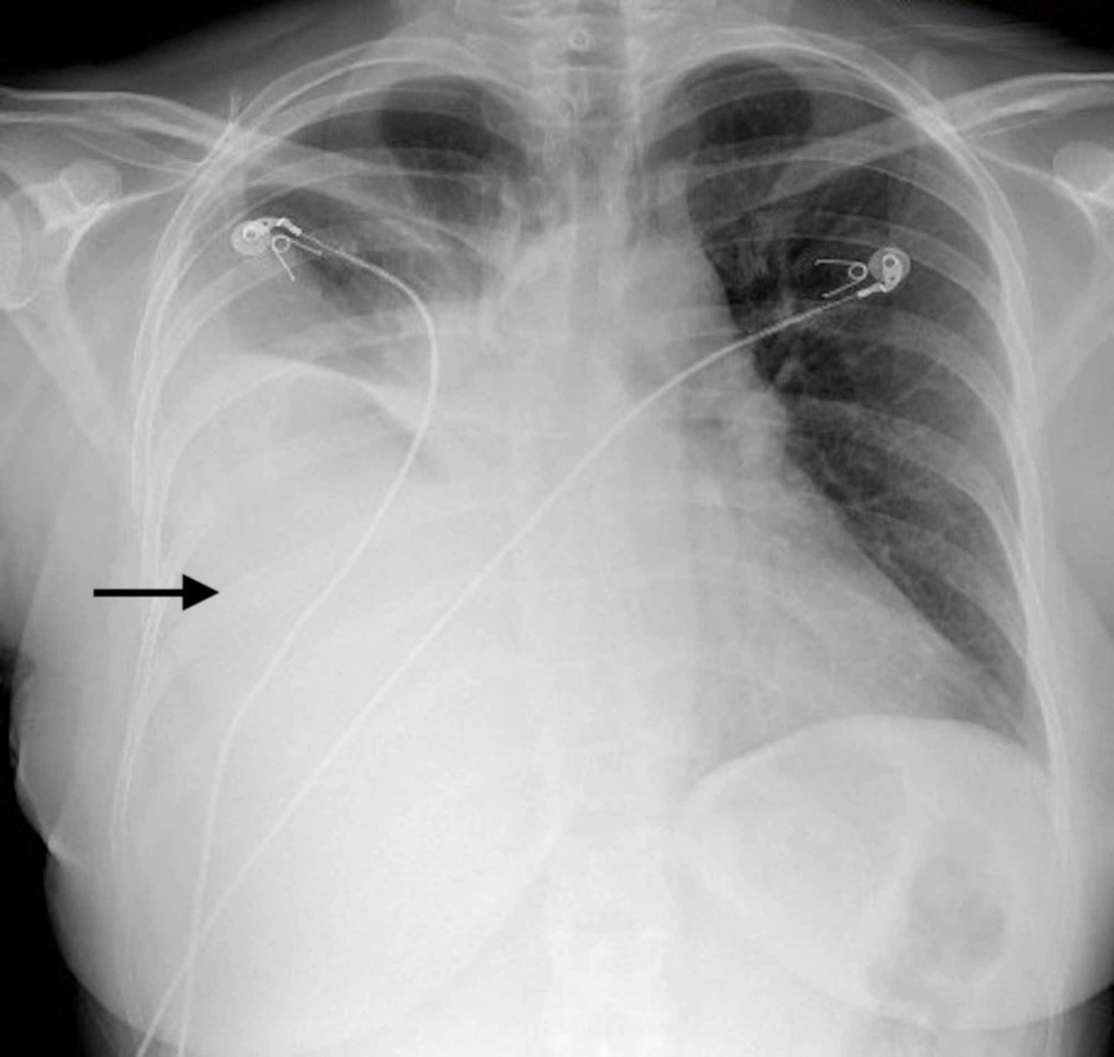
Chest X-ray with arrow pointing to a large right-sided pleural effusion

On laboratory testing alkaline phosphatase was elevated at 217 U/L, beta-hCG was elevated at 1,364 MIU/mL, arterial blood gas on 2 liters of oxygen showed no further hypoxemia, troponin levels were negative times two, and brain natriuretic peptide, basic chemistry panel, and complete blood counts were unremarkable. A chest X-ray showed a large right pleural effusion and suggested possible underlying atelectasis or infiltrate (Figure 1). Electrocardiogram showed non-specific ST segment changes and sinus tachycardia. Further workup included cancer antigen 125 (CA-125) elevated at 55.6 with follicle-stimulating hormone, and thyroid stimulating hormone levels within the normal limits. Novel coronavirus 2019, influenza nasal swabs, urine legionella and Streptococcus pneumoniae antigen testing were all negative. She was started on diuretic therapy and underwent thoracentesis with indwelling catheter drainage. A hemorrhagic exudative effusion was drained concerning for underlying malignancy. While awaiting pathology report the patient’s hCG remained elevated but consistent.

**Figure 2:**
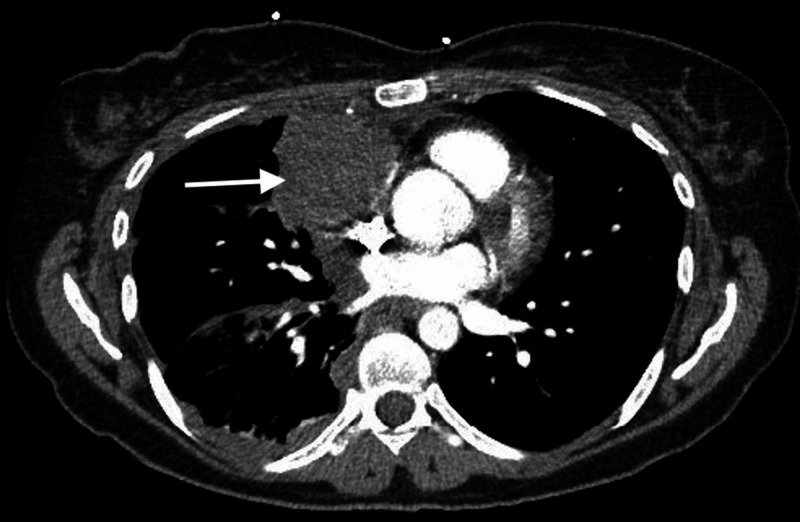
CT chest with arrow indicating right anterior mediastinal mass

Further imaging included a transvaginal ultrasound with no reported acute abnormalities. A computed tomography (CT) angiogram of the chest showed a soft tissue mass in the anterior medial right lung base/right middle lobe contacting the anterior mediastinum and anterior pleura measuring 5.0 x 4.8 cm (Figure 2). No pneumothorax or evidence of pulmonary embolism were identified. An abdominal and pelvis CT noted no acute abnormalities. Thoracentesis returned positive for cytokeratin-7, placental alkaline phosphatase, pankeratin, and GATA 3 consistent with malignant cells and favoring a germ cell tumor. A fiberoptic bronchoscopy did not identify any endobronchial lesions and washings were negative for malignancy. Subsequently a CT-guided biopsy of the aforementioned mediastinal mass was performed. In addition to the above stains, inhibin was weakly positive and both hCG and CD-10 were positive. Given the entire clinical scenario a diagnosis of primary choriocarcinoma was made. The patient’s breathing improved after pleural fluid drainage and she was discharged to follow up with a positron emission tomography (PET) scan and Oncologist as an outpatient.

## Discussion

PNMC is an extremely rare cause of a mediastinal mass. Establishment of the diagnosis relies on radiography followed by biopsy and histochemical staining. Tumor cells are strongly positive for syncytiotrophoblastic cells with hCG and CAM antigens [10]. As noted in Sicles et al., mediastinal choriocarcinoma is more aggressive and has a worse prognosis than gonadal choriocarcinoma [11]. Once identified patients should first be started on chemotherapeutic agents with consideration of adjuvant therapies. Nonseminomatous germ cell tumors are treated mainly with cisplatin-based chemotherapy regimens. A combination of Etoposide, ifosfamide, and cisplatin (VIP) is preferred over bleomycin, etoposide, and cisplatin (BEP) to avoid potential pulmonary side effects including acute respiratory distress syndrome and prolonged ventilation [12-14]. Following chemotherapy, residual tumors may benefit from surgical resection. A survival benefit was observed for nonseminomatous germ cell tumors, overall, that were found to have benign teratoma or necrosis on surgical pathology, with PNMC specifically showing survival benefit even with rising serum tumor markers [15, 16]. However, despite this combination, overall prognosis remains poor. Therapeutic options are limited in these unfortunate presentations as patients usually present late with non-specific symptomatology. Bokemeyer et al. reported a survival rate of 48% with combined chemotherapy and surgical intervention, but did not specify the percentage of PNMC [17]. In general for primary mediastinal nonseminomatous germ cell tumors, indicators of poor prognosis include age, viable tumor on excised masses, and resection in multiple locations [17, 18].

Our patient showed symptomatic improvement prior to discharge but will certainly need to maintain strict follow-up with oncology. Her effusion may reoccur, and if so, could be treated with repeat thoracentesis or indwelling pleural drainage. However, at this time the patient has not returned to our facility.

## Conclusions

Primary mediastinal choriocarcinoma is an uncommon cause of pleural effusion. This malignancy carries a high mortality and poor overall prognosis. Biopsy and immunohistochemical staining are crucial to establishing a diagnosis and, once obtained, chemotherapy along with adjunct therapies is the cornerstone of treatment.
